# Retrospective Analysis of Clinical Management Strategies for Cage Retropulsion Following Posterior Lumbar Interbody Fusion

**DOI:** 10.1111/os.70174

**Published:** 2025-09-24

**Authors:** Cheok‐Wa Iao, Xinhu Guo, Weipeng Qiu, Qiang Qi, Zhaoqing Guo, Chuiguo Sun, Woquan Zhong, Weishi Li

**Affiliations:** ^1^ Department of Orthopaedics Peking University Third Hospital Beijing China; ^2^ Beijing Key Laboratory of Spinal Disease Research Beijing China; ^3^ Engineering Research Center of Bone and Joint Precision Medicine, Ministry of Education Beijing China

**Keywords:** cage retropulsion, conservative treatment, lumbar spine revision surgery, posterior lumbar interbody fusion, reoperation

## Abstract

**Objectives:**

Cage retropulsion (CR) is a common complication following posterior lumbar interbody fusion (PLIF). Symptomatic patients with CR often require revision surgery. However, there is a lack of literature supporting the effectiveness of conservative treatment for CR. This study compares clinical and radiographic outcomes between conservative treatment and revision surgery in patients with CR after PLIF.

**Methods:**

A total of 55 patients with CR after PLIF treated at our institution between 2016 and 2023 were retrospectively reviewed; postoperative radiographic data of follow‐up were used to diagnose CR. Clinical outcomes were assessed before therapy and at the final follow‐up using the visual analog scale (VAS) for lower back pain and leg pain, Oswestry Disability Index (ODI) scores, and Japanese Orthopedic Association 29 (JOA‐29) scores. The treatment effectiveness was evaluated based on whether the score change reached the minimally clinically important difference (MCID). Radiographic indicators included the fusion rates, the extent of CR into the spinal canal, and the total displacement distance. Continuous variables were compared using independent samples *t*‐tests or Mann–Whitney *U* tests, while categorical variables were analyzed using Chi‐square or Fisher's exact tests, as appropriate. A *p*‐value < 0.05 was considered statistically significant.

**Results:**

The fusion rates at the final follow‐up for the conservative treatment group and the revision surgery group were 87.5% and 84.6%, respectively. There were no significant differences in final follow‐up fusion rates, lower back pain VAS scores, leg pain VAS scores, JOA scores, or ODI scores between the two groups (all *p* > 0.05). Additionally, there was no difference in the proportion of patients whose lower back pain VAS, ODI, and JOA scores achieved MCID between groups (all *p* > 0.05). However, in the revision surgery group, the proportion of patients whose leg VAS scores reached MCID was significantly higher than in the conservative group (*p* = 0.001). In the revision surgery subgroup analysis, patients who did not achieve leg VAS MCID demonstrated significantly more severe cage retropulsion distance compared to MCID achievers (*p* = 0.03).

**Conclusions:**

Conservative treatment yields satisfactory outcomes in mild, symptomatic CR patients, particularly for low back pain. For patients with a CR distance less than 8.8 mm, conservative treatment and revision surgery showed comparable outcomes, whereas when the CR distance is ≥ 8.8 mm, revision surgery was recommended to improve clinical results. Both conservative treatment and revision surgery can yield favorable outcomes when appropriately indicated.

## Graphical abstract

Conservative treatment achieved comparable outcomes to revision surgery in select cage retropulsion cases, with equivalent fusion rates, back pain visual analog scale (VAS), Japanese Orthopaedic Association (JOA), and ODI MCID attainment.

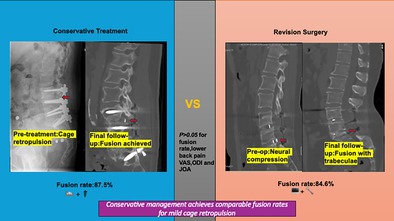

## 1 Introduction

Lumbar interbody fusion, an established treatment for a range of spinal disorders, is considered the “gold standard” surgical treatment for patients with degenerative lumbar diseases accompanied by lumbar segmental instability [1]. Compared to traditional posterolateral fusion, posterior lumbar interbody fusion (PLIF) provides a greater area for contact, increasing the rate of fusion [2, 3]; it also evacuates the disc, enlarging the neural foramina and reducing the probability of nerve root compression [4, 5]. However, PLIF and other operations are also associated with severe complications, such as pseudarthrosis, nerve injury, and adjacent segment disease [4], all of which represent major reasons for revision surgery.

Cage retropulsion (CR), also known as cage displacement after fusion surgery, is a rare complication after lumbar fusion surgery, and it is also the cause of lumbar revision surgery [3], which can directly compress the dural sac or nerve roots, cause a loss of lumbar lordosis, and result in significant neurological symptoms. Previous studies have extensively investigated the risk factors for CR following lumbar fusion surgery [3, 6, 7, 8, 9]. Although previous studies have confirmed the necessity of revision surgery for symptomatic CR, most studies involved small sample sizes and failed to focus on radiographic outcomes or the efficacy of conservative treatment [10, 11, 12].

In this study, data from patients who underwent revision surgery or conservative treatment for CR at our hospital over the past 7 years were retrospectively collected. This study aims to (i) evaluate the clinical and radiographic outcomes of conservative treatment versus revision surgery for CR following PLIF, (ii) investigate its underlying biomechanical mechanisms, and (iii) identify clinical indicators to guide optimal treatment selection.

## 2 Methods

### 2.1 Subjects

A total of 55 patients who experienced CR after PLIF for the degenerative lumbar disease at our hospital between January 2016 and December 2023 were analyzed. Among them, 39 were male and 16 were female, with an average age of 60.4 ± 13.5 years. Inclusion criteria were as follows: (1) Underwent primary PLIF for degenerative lumbar diseases; (2) postoperative follow‐up X‐ray or computed tomography (CT) scans showing the CR at lumbar level; (3) Complete minimum follow‐up of 12 months, with available imaging and clinical functional outcomes data. Exclusion criteria included: (1) Underwent lumbar surgery for non‐degenerative pathologies, such as trauma, tumors; (2) Surgical procedures other than PLIF, such as minimally invasive surgery, or simple discectomy; (3) follow‐up lasting less than 12 months or lack of imaging or clinical functional data at the final follow‐up; (4) cage fully migrated into the spinal canal.

According to the exclusion criteria, we excluded 10 cases (8 due to insufficient follow‐up duration and 2 due to complete cage migration into the spinal canal); a total of 55 cases were included in the study: 39 patients in the revision surgery group and 16 patients in the conservative treatment group.

### 2.2 Clinical and Radiological Evaluation

General demographic data, initial surgical details, and final follow‐up imaging and clinical functional scores (lower back pain and leg pain VAS scores, JOA‐29 scores, and ODI scores) were collected. The final follow‐up fusion rate, the improvement rate of each functional score, and the proportion of patients reaching the MCID were also assessed. The MCID values for each score were defined as follows: back pain VAS—1.21, leg pain VAS—1.28, ODI—11.79, and JOA—2.5 points [13].

All patients underwent imaging follow‐up at 1, 3, 6, and 12 months after the initial surgery, with at least 1 year follow‐up. Each patient had lumbar spine CT scans and anteroposterior and lateral X‐rays, as well as flexion‐extension X‐rays. Two physicians (with at least 5 years of clinical experience) collected the clinical data, and the average of their assessments was used. Independent discussions were held to reach a consensus regarding disputes regarding bone fusion judgment.

The following imaging diagnostic criteria were applied: CR was defined as the posterior edge of the cage reaching or partially entering (but not fully migrating into) the spinal canal on lumbar spine X‐rays or CT scans. Successful bone fusion was considered when clear, continuous trabeculae were observed on lumbar CT scans or when the range of motion in flexion‐extension X‐rays was less than five degrees.

### 2.3 Conservative Treatment

For patients who were initially diagnosed with CR after fusion surgery, if there were no significant symptoms of back or leg pain or if the symptoms were mild and not affecting daily life, conservative treatment was applied. The main conservative treatment measures included close outpatient follow‐up to monitor symptoms, limiting lumbar movement with a brace, and performing lumbar X‐rays or CT scans every 3 months.

**FIGURE 1:**
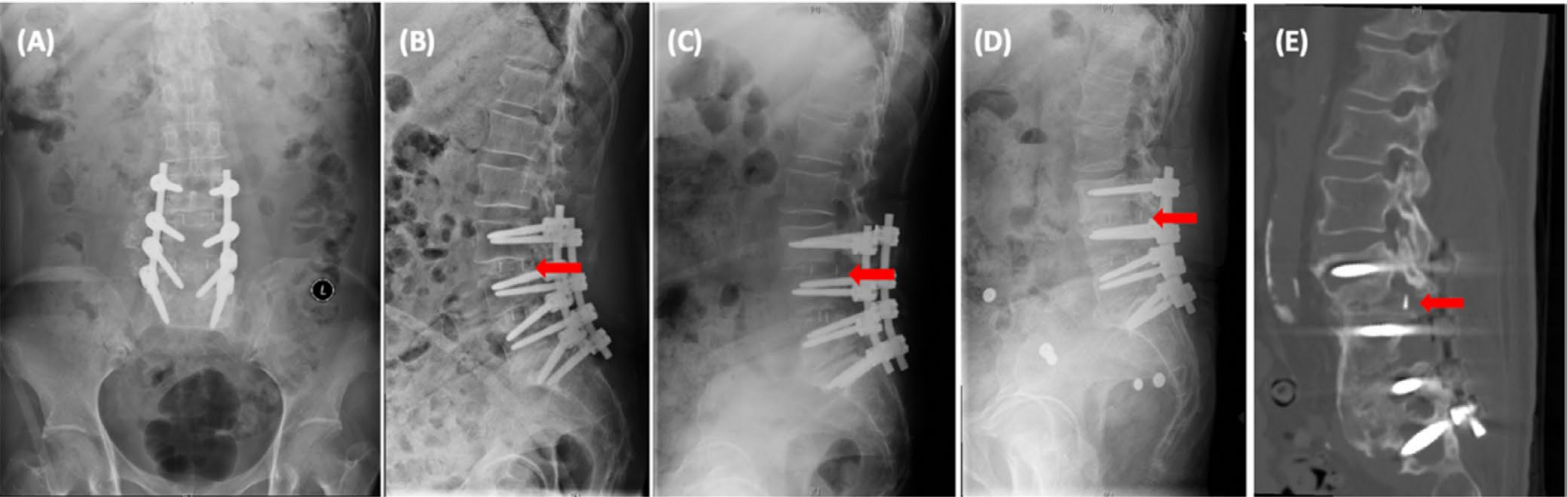
Images before and after conservative treatment for a female patient of 73 years old. (A) Postoperative anteroposterior radiogram. (B) Postoperative lateral radiogram. (C) The lateral radiogram at 3 months follow‐up shows the CR at L3/L4 level. (D) Lateral radiogram at 18 months follow‐up. (E) Sagittal reconstruction CT at L3/L4 level at final follow‐up, the patient's symptoms of lower back pain disappeared, and the CT indicated the successful fusion. The red arrow shows the posterior edge of the cage.

Symptomatic treatments included oral non‐steroidal anti‐inflammatory drugs (NSAIDs), local point injection blocks for pain, lumbar and back muscle exercises, physical therapy, or oral medications for osteoporosis. For patients with severe lower back or leg pain, failure of conservative treatment, or progressive CR during follow‐up, timely revision surgery was applied (Figure 1).

### 2.4 Revision Surgery Techniques

**FIGURE 2:**
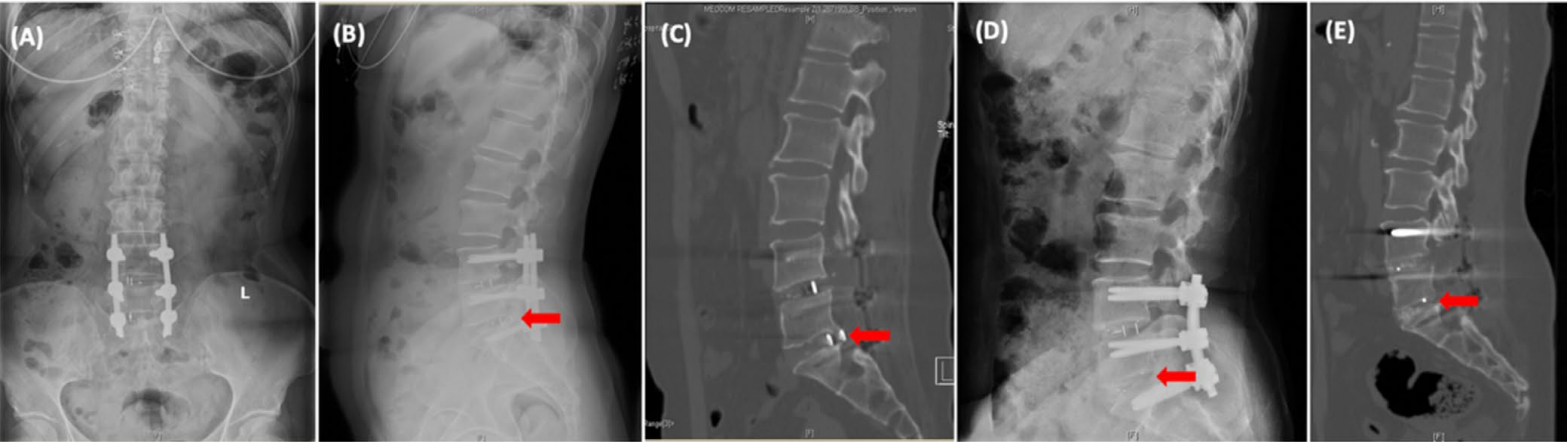
Images before and after revision surgery for a 53‐year‐old female patient. (A) Anteroposterior radiogram with the CR at L5/S1 before surgery. (B) Lateral radiogram with the CR at L5/S1 before revision surgery. (C) Sagittal reconstruction CT with the CR at L5/S1 before surgery. (D) Lateral radiogram after the revision surgery. The loose screws and cage were removed and replaced with larger ones, after which autologous bone grafts were re‐implanted for intervertebral fusion. (E) After the revision surgery, the final sagittal reconstruction CT at L5/S1 level showed that the patient's symptoms disappeared, and the CT indicated a successful fusion. The red arrow shows the posterior edge of the cage.

All patients who underwent revision surgery were treated using a posterior approach. The patient was placed in a prone position, and the original incision was reopened to expose the bilateral pedicles. Blunt dissection was performed to remove the extensive scar tissue and paraspinal muscles surrounding the original cage. In 27 patients, the displaced cage was removed, and after preparing the interbody bone graft bed, a new cage of appropriate size filled with autologous bone fragments was inserted, along with allograft bone and bone morphogenetic protein (BMP). In 8 patients, the displaced cage was removed, but no new cage was inserted; in four patients, the displaced cage was not removed but was repositioned anteriorly. After appropriately compressing the interbody space and locking it, the stability of the cage was confirmed, and the wound was closed layer by layer (Figure 2).

In the revision cases mentioned above, nine patients underwent transverse process decortication followed by fusion using allograft or autologous bone fragments. Posterolateral bone grafting fusion was performed in five patients. For the management of loose screws, 17 patients underwent impaction bone grafting, 3 patients had screws revised with bone cement augmentation, 9 patients had the original screws replaced with larger screws for fixation, and 7 patients underwent new screw tract puncturing. Additionally, two patients had S2AI screw fixation performed with the assistance of the Medtronic Mazor X robotic system. The cages used in these procedures were either kidney‐shaped or rectangular, featuring a hollow bone graft window, and made of polyether‐ether‐ketone (PEEK).

### 2.5 Statistical Analysis

SPSS 26.0 (IBM Corp., USA) was used for data analysis. Continuous variables were expressed as mean ± standard deviation, while categorical variables were presented as frequencies or percentages. Independent sample *t*‐tests or Mann–Whitney U tests were used to analyze continuous variables, and chi‐square or Fisher's exact tests were employed for categorical variables. Comparisons were made between the two groups regarding demographic data, clinical scores, and imaging parameters before and after treatment. A subgroup analysis was performed based on whether patients in the revision surgery group achieved the leg VAS MCID, followed by intergroup comparisons using univariate analysis. A *p*‐value < 0.05 was considered statistically significant.

## 3 Results

### 3.1 Patient Demographics

The revision group comprised 39 cases, consisted of 28 males and 11 females, with an average age of 59 years (range, 19–79) and an average follow‐up period of 27 months (range, 12–89 months); the time of CR detection postoperatively was 3.7 months (range, 1–12 months). The conservative group included 16 cases; there were 11 males and 5 females, with an average age of 63 years (range, 22–91) and an average follow‐up period of 37 months (range, 13–80 months); the time of CR detection postoperatively was 3.2 months (range, 3–6 months). There were no significant differences between the conservative and revision groups regarding age, gender, body mass index (BMI), smoking history, follow‐up duration, type of primary disease, osteoporosis, or the time of CR detection postoperatively (all *p* > 0.05). The specific univariate analysis results for both groups are shown in Table 1.

**TABLE 1:** Comparison of baseline data between the conservative treatment group and revision surgery group [*N* (%), *M* ± SD].

	Total cases	Conservative treatment group	Revision surgery group	*t*/*X* ^2^	*p*
Gender				0.051	0.821
Male	39 (70.9)	11 (28.2)	28 (71.8)		
Female	16 (29.1)	5 (31.3)	11 (68.8)		
Diagnosis				2.129	0.345
Disc herniations	26 (47.3)	10 (38.5)	16 (61.5)		
Lumbar stenosis	20 (36.4)	4 (20.0)	16 (80.0)		
Spondylolisthesis	9 (16.4)	2 (22.2)	7 (77.8)		
Smoking history				0.076	0.783
Yes	8 (14.5)	2 (25.0)	6 (75.0)		
No	47 (85.5)	14 (29.8)	33 (70.2)		
Osteoporosis				3.851	0.050
Yes	17 (30.9)	8 (47.1)	9 (52.9)		
No	38 (69.1)	8 (21.1)	30 (78.9)		
Age (years)	60.4 ± 13.5	62.7 ± 17.0	59.4 ± 11.8	0.812	0.420
BMI (kg/m^2^)	25.7 ± 3.8	26.8 ± 4.5	25.3 ± 3.4	1.364	0.178
Follow‐up time (months)	29.7 ± 21.8	37.0 ± 21.7	26.6 ± 21.5	1.621	0.111
First operation time (min)	169.0 ± 52.0	164.1 ± 34.4	171.4 ± 59.0	−0.446	0.658
First operation blood loss (mL)	502.7 ± 360.0	519.3 ± 239.4	495.4 ± 404.9	0.213	0.832
Discover CR time after surgery	3.6 ± 2.9	3.2 ± 0.8	3.7 ± 3.4	−0.597	0.553
The interval between CR and revision	2.6 ± 5.5	1.1 ± 3.8	3.2 ± 6.0	−1.265	0.211

### 3.2 Clinical Outcomes

The pre‐treatment leg VAS, JOA, and ODI scores in the revision group were significantly worse than those in the conservative group (all *p* = 0.001), whereas no significant intergroup differences were observed in these scores at the final follow‐up, nor in the proportion of patients achieving MCID (all *p* > 0.05). Although the revision group yielded significantly greater improvements in leg VAS, JOA, and ODI scores than the conservative group (all *p* < 0.05), only the proportions of patients achieving MCID for leg VAS showed statistically significant superiority. Additionally, there were no significant differences between the revision and conservative groups regarding lower back pain VAS scores before and after treatment, the pre‐ and post‐treatment score differences, or the proportion of patients achieving the MCID (all *p* > 0.05).

Among the 39 patients who underwent revision surgery at our hospital, 5 experienced a small cerebrospinal fluid leak during surgery, 1 patient had an injury to the left L5 nerve root, 1 patient experienced worsening leg pain postoperatively, 1 patient had no improvement in leg pain, 1 patient showed a worsening in ODI score, and 1 patient had a worsening in JOA score. Additioinal clinical data are presented in Table 2.

**TABLE 2:** Comparison of clinical data between conservative treatment group and revision surgery group [*N* (%), *M* ± SD].

	Total cases	Conservative treatment group	Revision surgery group	*t*/*X* ^2^	*p*
Leg VAS score (before treatment)	4.8 ± 2.6	2.3 ± 2.3	5.8 ± 2.0	−5.633	0.001
Leg VAS score (after treatment)	1.0 ± 1.7	0.6 ± 1.2	1.2 ± 1.9	−1.213	0.231
Leg VAS difference	3.8 ± 2.7	1.8 ± 2.1	4.6 ± 2.4	−4.139	0.001
Back VAS difference (before treatment)	4.4 ± 1.5	3.9 ± 1.6	4.6 ± 1.4	−1.547	0.128
Back VAS difference (after treatment)	1.6 ± 1.6	1.3 ± 2.0	1.7 ± 1.5	−0.852	0.398
Back VAS difference	2.8 ± 1.7	2.6 ± 1.6	2.9 ± 1.8	−0.529	0.599
JOA score (before treatment)	15.4 ± 6.2	20.5 ± 5.6	13.2 ± 5.1	4.635	0.001
JOA score (after treatment)	24.4 ± 4.5	26.1 ± 3.0	23.7 ± 4.9	1.817	0.075
JOA difference	9.0 ± 6.7	5.6 ± 6.5	10.4 ± 6.2	−2.596	0.012
ODI score (before treatment)	21.7 ± 10.3	14.6 ± 9.1	24.6 ± 9.4	−3.704	0.001
ODI score (after treatment)	5.5 ± 7.6	4.8 ± 7.5	5.7 ± 7.8	−0.407	0.685
ODI difference	16.2 ± 10.9	9.8 ± 6.8	18.9 ± 11.3	−3.021	0.002
Leg VAS_MCID				8.305	0.004
Yes	43 (78.2)	8 (18.6)	35 (81.4)		
No	12 (21.8)	8 (66.7)	4 (33.3)		
Back VAS_MCID				2.159	0.142
Yes	45 (81.8)	15 (33.3)	30 (66.7)		
No	10 (18.2)	1 (10.0)	9 (90.0)		
JOA_MCID				1.190	0.275
Yes	40 (72.7)	10 (25.0)	30 (75.0)		
No	15 (27.3)	6 (40.0)	9 (60.0)		
ODI_MCID				2.383	0.123
Yes	36 (65.5)	8 (22.2)	28 (77.8)		
No	19 (34.5)	8 (42.1)	11 (57.9)		

### 3.3 Radiographic Parameters

According to radiographic assessment, the final fusion rate was 84.6% (33/39) in the revision group and 87.5% (14/16) in the conservative group, with no significant difference between the two groups (*p* = 0.783). Similarly, no significant intergroup differences were observed in the number of cage displacements or endplate injuries (all *p* > 0.05). In the revision group, 35 patients had a single CR, while 4 patients had two cages retropulsed. In the conservative group, all 16 patients had only one cage displaced. The number of patients with CR at the L3/L4 segment was significantly higher in the conservative group than in the revision group (*p* = 0.014). It is noteworthy that the CR distance showed no statistically significant differences across three time points: initial diagnosis (*p* = 0.085), pre‐treatment evaluation (*p* = 0.050) and final follow‐up (*p* = 0.676), while the total CR displacement distance also exhibited no intergroup differences (*p* = 0.197). However, the revision group demonstrated greater immediate post‐operative cage migration distance than the conservative group (*p* = 0.04), whereas it exhibited significantly less intraspinal displacement distance of the cage (*p* = 0.001). The specific radiographic parameters are shown in Table 3.

**TABLE 3:** Comparison of radiographic parameters between the conservative treatment group and revision surgery group [*N* (%), *M* ± SD].

	Total cases	Conservative treatment group	Revision surgery group	*t*/*X* ^2^	*p*
L1 CT value	130.8 ± 42.6	124.5 ± 55.1	133.4 ± 36.7	−0.694	0.491
Endplate injury				0.000	0.991
Yes	24 (43.6)	7 (29.2)	17 (70.8)		
No	31 (56.4)	9 (29.0)	22 (71.0)		
Retropulsion number					0.3111^†^
1	51 (92.7)	16 (31.4)	35 (68.6)		
2	4 (7.3)	0 (0.0)	4 (100.0)		
Retropulsion level				14.346	0.014
L1–L2	1 (1.8)	1 (100.0)	0 (0.0)		
L2–L3	2 (3.6)	1 (50.0)	1 (50.0)		
L3–L4	6 (10.9)	5 (83.3)	1 (16.7)		
L3–L5	4 (7.3)	0 (0.0)	4 (100.0)		
L4–L5	16 (29.1)	3 (18.8)	13 (81.3)		
L5–S1	26 (47.3)	6 (23.1)	20 (76.9)		
Last follow‐up fusion rate				0.076	0.783
Yes	47 (85.5)	14 (29.8)	33 (70.2)		
No	8 (14.5)	2 (25.0)	6 (75.0)		
Immediate cage position after surgery	8.6 ± 4.1	10.35 ± 3.6	7.9 ± 4.1	2.215	0.034
Initial CR position	8.5 ± 4.4	6.9 ± 4.7	9.2 ± 4.2	−1.671	0.107
Pre‐treatment CR position	8.9 ± 4.5	6.9 ± 4.7	9.7 ± 4.2	−2.059	0.050
The last follow‐up CR position	9.6 ± 4.3	9.2 ± 4.6	9.7 ± 4.2	−0.420	0.676
Whole cage migration distance (mm)	17.9 ± 5.8	19.5 ± 7.0	17.3 ± 5.2	1.305	0.197
Cage migration distance intraspinal	1.0 ± 1.6	2.3 ± 2.0	0.5 ± 1.1	4.217	0.001

^†^ Fisher's exact test.

### 3.4 Subgroup Analysis Results

Within the revision surgery group, subgroup analysis was performed based on the achievement of the leg VAS MCID, with displacement severity dichotomized at the median(8.82 mm). Results demonstrated that patients who achieved the leg VAS MCID had a significantly higher proportion of mild CR displacement (< 8.82 mm) compared to non‐achievers (*p* = 0.03). No significant intergroup differences were observed in age, baseline VAS scores (≥ 7), or endplate injury status (all *p* > 0.05). The detailed results for both subgroups are presented in Table 4. Additional subgroup analyses stratified by the achievement of MCID thresholds for back VAS, leg VAS, JOA, and ODI scores revealed no significant differences in CR distance or fusion rates (all *p* > 0.05).

**TABLE 4:** Comparison of baseline data between the revision group achieving leg VAS MCID and not achieving leg VAS MCID [*N* (%), *M* ± SD].

	Total cases	Achieving leg VAS MCID group	Not achieving leg VAS MCID group	*t*/*X* ^2^	*p*
Age (years)				0.803	0.370
< 60	18 (46.2)	1 (5.6)	17 (94.4)		
≥ 60	21 (53.8)	3 (14.3)	18 (85.7)		
CR displacement (mm)				4.692	0.030
< 8.82	20 (51.3)	0 (0.0)	20 (100.0)		
≥ 8.82	19 (48.7)	4 (21.1)	15 (78.9)		
VAS				0.341	0.559
< 7	24 (61.5)	3 (12.5)	21 (87.5)		
≥ 7	15 (38.5)	1 (6.7)	14 (93.3)		
Endplate injury				1.788	0.181
Yes	17 (43.6)	3 (17.6)	14 (82.4)		
No	22 (56.4)	1 (4.5)	21 (95.5)		

## 4 Discussion

### 4.1 Summary of Key Findings

Current consensus in the literature suggests that conservative treatment is appropriate for asymptomatic CR patients, while revision surgery is mandatory for CR patients presenting with neurological symptoms [3, 6, 7, 8, 9, 14]. However, our findings demonstrate that selected symptomatic CR patients may achieve favorable clinical outcomes through conservative treatment. In this study, the pre‐treatment degree of CR in the conservative group was smaller than that in the revision group (6.9 vs. 9.7 mm), although the intergroup difference was not statistically significant (*p* > 0.05). Notably, both groups ultimately achieved satisfactory radiographic fusion at the final follow‐up. The revision group exhibited more severe baseline clinical scores (except for lower back VAS) compared to the conservative group. However, according to our findings, the revision surgery achieved significantly better MCID achievement rates only for leg VAS scores compared to conservative treatment, with no differences observed in ODI, JOA, or lower back VAS outcomes (*p* > 0.05). Interestingly, among the 8 patients in the conservative group who failed to achieve MCID for leg VAS, we found that 2 cases had a baseline VAS score of only 1 point, 5 cases had 0 points (no pain at baseline), and only 1 case showed no improvement in leg pain.

### 4.2 Mechanistic Analyses

The etiology of cage retropulsion can be explained through biomechanical mechanisms, primarily involving the stability of the bone–implant interface. Multiple studies have emphasized that intraoperative endplate injury plays a key role for cage retropulsion [15], as it reduces friction and axial compressive forces between the endplate and interbody cage, consequently diminishing intervertebral load‐bearing capacity and predisposing to CR. In our patient cohort, the majority had no endplate injury preoperatively (31 patients, 56.4%). Subgroup analysis based on the presence or absence of endplate injury revealed no significant differences in the final fusion rates or clinical outcomes between groups (all *p* > 0.05). Concurrently, adequate posterior fixation strength is critical for preventing CR, as it substantially enhances axial compression stiffness while reducing posterior bending forces [7, 16, 17]. Numerous studies have identified screw loosening as an independent risk factor for CR, with osteoporosis‐induced reduction in trabecular bone density frequently leading to diminished screw fixation strength and impaired bone fusion, thereby contributing to CR [7, 18]. Our cohort consisted predominantly of non‐osteoporotic patients (38, 69%). However, the revision surgery group showed a higher proportion of osteoporotic patients compared to the conservative treatment group, although the difference did not reach statistical significance (*p* = 0.05). Subgroup analysis based on osteoporosis status revealed no significant differences in final fusion rates or clinical outcome scores between groups (all *p* > 0.05). Current consensus recommends anterior positioning of the cage, as anterior placement generates greater longitudinal stress and prevents CR [7, 19, 20, 21]. Additionally, Yang et al. reported that pear‐shaped intervertebral discs increase the risk of CR. The underlying mechanism suggests that the anteriorly narrow and posteriorly wide morphology of pear‐shaped discs predisposes to posterior migration of partially ununited cages. Therefore, achieving optimal cage–endplate congruency and maintaining sufficient, evenly distributed longitudinal tension are crucial for ensuring early cage stability [7, 22].

### 4.3 The Critical Role of CR Distance in Our Findings

Subgroup analysis of the revision surgery cohort revealed that patients who achieved the leg VAS MCID had a significantly higher proportion of mild CR displacement (< 8.8 mm) compared to those with severe displacement (*p* = 0.03). Although logistic regression analysis did not show statistical significance, our findings suggest: For CR displacement < 8.8 mm, conservative treatment and revision surgery demonstrate comparable clinical outcomes. For CR displacement ≥ 8.8 mm, revision surgery is the recommended therapeutic approach. Therefore, we believe conservative treatment can achieve satisfactory outcomes and fusion rates for patients with mild clinical symptoms and less severe cage displacement, whereas revision surgery should be reserved for cases with severe neurological compromise or progressive symptoms. Masato et al. [20] proposed a novel classification system for CR and argued that revision surgery is not necessary for patients with only back pain due to CR, recommending surgery primarily for those with lower limb symptoms. The results of our study are consistent with their findings, particularly for patients with predominantly back pain, where there was no significant difference in the degree of improvement and efficacy between the two treatment methods (*p* > 0.05). However, their study included only 5 CR cases and did not report postoperative outcomes following revision procedures. Pan et al. [6, 23] reported surgical cases of CR patients who underwent revision procedures, though their study was similarly limited by a small sample size (*n* = 6). Zhang et al. [24, 25] reported successful cases of conservative treatment in 10 patients with CR. Similarly, Lee et al. [17] suggested that some patients with back and leg pain may not require revision surgery. Our study further validates the efficacy of conservative treatment for symptomatic CR patients. However, revision surgery may still offer superior advantages for leg pain relief compared to conservative treatment. It is important to note that the studies above did not specifically compare the efficacy of the two treatment approaches, and research on the effectiveness of conservative treatment remains limited.

### 4.4 Technical Challenges and Considerations in Revision Surgery

Revision surgery for cage removal via the posterior approach presents several technical challenges. Scar tissue from the previous surgery can obscure normal anatomical landmarks, making intraoperative navigation difficult and increasing the risk of dural retraction and nerve injury [26, 27]. Additionally, some studies [27, 28] have indicated that re‐injury to the paraspinal muscles and ligaments during posterior revision increases the risk of post‐operative infection, pain, and bleeding by 15% to 30%. Selznick et al. [26] also discussed using minimally invasive PLIF in revision surgeries but noted an increased risk of cerebrospinal fluid leakage. Zhang et al. [29] suggested that using an O‐arm surgical navigation system effectively improves the accuracy and success rate of lumbar revision surgeries. Among the 29 patients who underwent revision surgery in this study, 5 experienced intraoperative complications involving dural or nerve injuries (17%), a rate similar to that reported in previous studies [30]. Additionally, 4 patients experienced worsening or no improvement in leg pain postoperatively. These patients showed some symptom improvement within 3 months with conservative symptomatic treatment, and no further surgery was required. All other patients experienced significant improvement in their scores after revision surgery. Based on our experience, the fundamental principles for CR revision surgery are decompression and stability reconstruction: first, the displaced cage should be removed and replaced with a larger cage along with autologous or allograft bone; second, stability should be ensured by replacing loose screws or extending the fixation segment; third, scar tissue during surgery should be carefully dissected to avoid nerve damage.

### 4.5 Clinical Implications and Limitations of the Study

This study provides clinical evidence comparing treatment outcomes between conservative management and revision surgery for CR after PLIF. In our comparative analysis of patients with similar pre‐treatment CR severity, we found that for patients with a small retropulsion distance into the spinal canal and clinical symptoms, conservative treatment may still achieve satisfactory outcomes and fusion rates. To the best of our knowledge, this is the first study that directly compared the efficacy of revision surgery and conservative treatment for CR following PLIF. This represents one of the largest single‐center series reporting both radiographic fusion rates and clinical outcomes after posterior revision for CR. The present study has several limitations. First, due to the low incidence of CR, the sample size remains relatively small, with 55 cases. Second, this is a retrospective study, lacking randomization and intervention control. Third, we did not analyze the role of sagittal spinal parameters in the outcomes. Future research with larger sample sizes and prospective studies is needed to provide stronger evidence.

## 5 Conclusions

In conclusion, our findings refine the treatment paradigm for symptomatic CR by integrating clinical outcomes with biomechanical insight. We demonstrate that the decision between conservative treatment and revision surgery should be guided by the degree of cage displacement and the nature of symptoms, rather than the mere presence of symptoms. Specifically, a displacement threshold of 8.8 mm emerges as a key radiographic indicator. We therefore recommend an initial trial of non‐operative treatment for patients with mild displacement (< 8.8 mm) and non‐radicular pain, while advocating for expeditious surgical intervention in those with severe displacement (≥ 8.8 mm) or significant neurological compromise. With properly selected indications, conservative treatment and revision surgery can lead to good clinical outcomes.

## Author Contributions

All authors had full access to the data in the study and take responsibility for the integrity of the data and the accuracy of the data analysis. Conceptualization, C.‐W.I., X.G. and W.L.; Methodology, C.‐W.I. and X.G.; Validation, W.Q. and X.G.; Investigation, C.‐W.I.; Formal Analysis, C.‐W.I. and X.G.; Resources, Q.Q., Z.G., C.S. and W.Z.; Original Draft, C.‐W.I.; Review and Editing, X.G. and W.L.; Visualization, C.‐W.I. and X.G.; Supervision, X.G. and W.L.

## Disclosure

The authors have nothing to report.

## Ethics Statement

All procedures performed in studies were in accordance with principles of the Declaration of Helsinki. The study was approved by the Institutional Review Board of the Medical Science Research Ethics Committee of Peking University Third Hospital.

## Consent

Written informed consent was obtained from the patient for any data and accompanying images of this research.

## Conflicts of Interest

The authors declare no conflicts of interest.

## Data Availability

The data that support the findings of this study are available from the corresponding author upon reasonable request.

## References

[1] R. J. Mobbs , K. Phan , G. Malham , K. Seex , and P. J. Rao , “Lumbar Interbody Fusion: Techniques, Indications and Comparison of Interbody Fusion Options Including PLIF, TLIF, MI‐TLIF, OLIF/ATP, LLIF and ALIF,” Journal of Spine Surgery 1, no. 1 (2015): 2–18. 27683674 10.3978/j.issn.2414-469X.2015.10.05 PMC5039869

[2] I. Teng , J. Han , K. Phan , and R. Mobbs , “A Meta‐Analysis Comparing ALIF, PLIF, TLIF and LLIF,” Journal of Clinical Neuroscience 44 (2017): 11–17. 28676316 10.1016/j.jocn.2017.06.013

[3] H. Kimura , J. Shikata , S. Odate , T. Soeda , and S. Yamamura , “Risk Factors for Cage Retropulsion After Posterior Lumbar Interbody Fusion: Analysis of 1070 Cases,” Spine (Phila Pa 1976) 37, no. 13 (2012): 1164–1169. 22647991 10.1097/BRS.0b013e318257f12a

[4] K. Liu , H. Chang , L. Wang , C. Wang , T. Chen , and X. Meng , “Risk Factors for Cage Retropulsion After Lumbar Interbody Fusion: Systematic Review and Meta‐Analysis,” World Neurosurgery 132 (2019): 273–281. 31521758 10.1016/j.wneu.2019.09.019

[5] M. Madera , J. Brady , S. Deily , et al., “The Role of Physical Therapy and Rehabilitation After Lumbar Fusion Surgery for Degenerative Disease: A Systematic Review,” Journal of Neurosurgery. Spine 26, no. 6 (2017): 694–704. 28291412 10.3171/2016.10.SPINE16627

[6] F. M. Pan , S. J. Wang , Z. Y. Yong , X. M. Liu , Y. F. Huang , and D. S. Wu , “Risk Factors for Cage Retropulsion After Lumbar Interbody Fusion Surgery: Series of Cases and Literature Review,” International Journal of Surgery 30 (2016): 56–62. 27107661 10.1016/j.ijsu.2016.04.025

[7] Y. Hou , H. Shi , H. Shi , T. Zhao , J. Shi , and G. Shi , “A Meta‐Analysis of Risk Factors for Cage Migration After Lumbar Fusion Surgery,” World Neurosurg X 18 (2023): 100152. 36785623 10.1016/j.wnsx.2023.100152 PMC9918778

[8] H. Li , H. Wang , Y. Zhu , W. Ding , and Q. Wang , “Incidence and Risk Factors of Posterior Cage Migration Following Decompression and Instrumented Fusion for Degenerative Lumbar Disorders,” Medicine (Baltimore) 96, no. 33 (2017): e7804. 28816975 10.1097/MD.0000000000007804 PMC5571712

[9] L. Peng , J. Guo , J. P. Lu , S. Jin , P. Wang , and H. Y. Shen , “Risk Factors and Scoring System of Cage Retropulsion After Posterior Lumbar Interbody Fusion: A Retrospective Observational Study,” Orthopaedic Surgery 13, no. 3 (2021): 855–862. 33749137 10.1111/os.12987 PMC8126950

[10] R. Bassani , C. Morselli , A. Cirullo , A. M. Querenghi , and L. Mangiavini , “Successful Salvage Strategy Using Anterior Retroperitoneal Approach in Failed Posterior Lumbar Interbody Fusion. A Retrospective Analisys on Lumbar Lordosis and Clinical Outcome,” European Spine Journal 31, no. 7 (2022): 1649–1657. 35652952 10.1007/s00586-022-07247-2

[11] A. R. D. Pereira Filho , V. S. Baptista , M. Mussalem , et al., “Reoperations for Cage Removal or Replacement in Patients Undergoing ALIF: Operative Morbidity and Surgical Strategy,” Neurosurgical Review 48, no. 1 (2025): 499. 40493101 10.1007/s10143-025-03670-3

[12] W. Wang , J. Li , Y. Xu , Y. Luo , W. Ding , and W. Zhang , “Predictors and Tactics for Revision Surgery in Lateral Lumbar Interbody Fusion,” BMC Musculoskeletal Disorders 23, no. 1 (2022): 1101. 36528567 10.1186/s12891-022-06052-8 PMC9758827

[13] L. Y. Carreon , K. R. Bratcher , C. E. Canan , L. O. Burke , M. Djurasovic , and S. D. Glassman , “Differentiating Minimum Clinically Important Difference for Primary and Revision Lumbar Fusion Surgeries,” Journal of Neurosurgery. Spine 18, no. 1 (2013): 102–106. 23157276 10.3171/2012.10.SPINE12727

[14] M. Moisi , J. Page , D. Paulson , and R. J. Oskouian , “Technical Note ‐ Lateral Approach to the Lumbar Spine for the Removal of Interbody Cages,” Cureus 7, no. 5 (2015): e268. 26180692 10.7759/cureus.268 PMC4494582

[15] Y. Yu , D. L. Robinson , D. C. Ackland , Y. Yang , and P. V. S. Lee , “Influence of the Geometric and Material Properties of Lumbar Endplate on Lumbar Interbody Fusion Failure: A Systematic Review,” Journal of Orthopaedic Surgery and Research 17, no. 1 (2022): 224. 35399075 10.1186/s13018-022-03091-8 PMC8996478

[16] N. Li , M. Dai , B. Zhang , et al., “Risk Factors for Cage Retropulsion After Transforaminal Lumbar Interbody Fusion in Older Patients,” Annals of Translational Medicine 8, no. 24 (2020): 1660. 33490172 10.21037/atm-20-7416 PMC7812186

[17] D. Y. Lee , Y. J. Park , S. Y. Song , S. T. Jeong , and D. H. Kim , “Risk Factors for Posterior Cage Migration After Lumbar Interbody Fusion Surgery,” Asian Spine J 12, no. 1 (2018): 59–68. 29503683 10.4184/asj.2018.12.1.59 PMC5821934

[18] Z. J. Zhou , P. Xia , F. D. Zhao , X. Q. Fang , S. W. Fan , and J. F. Zhang , “Endplate Injury as a Risk Factor for Cage Retropulsion Following Transforaminal Lumbar Interbody Fusion: An Analysis of 1052 Cases,” Medicine (Baltimore) 100, no. 5 (2021): e24005. 33592856 10.1097/MD.0000000000024005 PMC7870182

[19] Y. H. Hu , C. C. Niu , M. K. Hsieh , T. T. Tsai , W. J. Chen , and P. L. Lai , “Cage Positioning as a Risk Factor for Posterior Cage Migration Following Transforaminal Lumbar Interbody Fusion ‐ An Analysis of 953 Cases,” BMC Musculoskeletal Disorders 20, no. 1 (2019): 260. 31142310 10.1186/s12891-019-2630-0 PMC6542074

[20] M. Tanaka , Z. Wei , A. Kanamaru , et al., “Revision for Cage Migration After Transforaminal/Posterior Lumbar Interbody Fusion: How to Perform Revision Surgery?,” BMC Surgery 22, no. 1 (2022): 172. 35546229 10.1186/s12893-022-01620-0 PMC9092779

[21] W. Singhatanadgige , A. Sukthuayat , T. Tanaviriyachai , et al., “Risk Factors for Polyetheretherketone Cage Subsidence Following Minimally Invasive Transforaminal Lumbar Interbody Fusion,” Acta Neurochirurgica 163, no. 9 (2021): 2557–2565. 34297205 10.1007/s00701-021-04923-y

[22] W. Schmoelz and A. Keiler , “Intervertebral Cages From a Biomechanical Point of View,” Der Orthopäde 44, no. 2 (2015): 132–137. 25595216 10.1007/s00132-014-3071-y

[23] Y. Aoki , M. Yamagata , F. Nakajima , Y. Ikeda , and K. Takahashi , “Posterior Migration of Fusion Cages in Degenerative Lumbar Disease Treated With Transforaminal Lumbar Interbody Fusion: A Report of Three Patients,” Spine (Phila Pa 1976) 34, no. 1 (2009): E54–E58. 19127150 10.1097/BRS.0b013e3181918aae

[24] M. Zhang , X. Liu , G. Wang , H. Liu , F. Zhu , and H. Mou , “Risk Factors Associated With Cage Retropulsion After Lumbar Interbody Fusion,” Turkish Neurosurgery 34, no. 2 (2024): 274–282. 37614214 10.5137/1019-5149.JTN.43124-23.2

[25] L. Chen , H. Yang , and T. Tang , “Cage Migration in Spondylolisthesis Treated With Posterior Lumbar Interbody Fusion Using BAK Cages,” Spine (Phila Pa 1976) 30, no. 19 (2005): 2171–2175. 16205342 10.1097/01.brs.0000180402.50500.5b

[26] L. A. Selznick , M. F. Shamji , and R. E. Isaacs , “Minimally Invasive Interbody Fusion for Revision Lumbar Surgery: Technical Feasibility and Safety,” Journal of Spinal Disorders & Techniques 22, no. 3 (2009): 207–213. 19412024 10.1097/BSD.0b013e318169026f

[27] C. H. Kim , C. K. Chung , T. A. Jahng , H. J. Yang , and Y. J. Son , “Surgical Outcome of Percutaneous Endoscopic Interlaminar Lumbar Diskectomy for Recurrent Disk Herniation After Open Diskectomy,” Journal of Spinal Disorders & Techniques 25, no. 5 (2012): E125–E133. 22744610 10.1097/BSD.0b013e31825bd111

[28] K. M. Eichholz and T. C. Ryken , “Complications of Revision Spinal Surgery,” Neurosurgical Focus 15, no. 3 (2003): E1. 10.3171/foc.2003.15.3.1 15347219

[29] W. Zhang , T. Takigawa , Y. Wu , Y. Sugimoto , M. Tanaka , and T. Ozaki , “Accuracy of Pedicle Screw Insertion in Posterior Scoliosis Surgery: A Comparison Between Intraoperative Navigation and Preoperative Navigation Techniques,” European Spine Journal 26, no. 6 (2017): 1756–1764. 28028647 10.1007/s00586-016-4930-5

[30] K. Kobayashi , K. Ando , F. Kato , et al., “Reoperation Within 2 Years After Lumbar Interbody Fusion: A Multicenter Study,” European Spine Journal 27, no. 8 (2018): 1972–1980. 29423887 10.1007/s00586-018-5508-1

